# Enzymatic Extraction of Hawthorn Pectin Under Ultrahigh Pressure: Optimization and Characterization

**DOI:** 10.3390/molecules30102210

**Published:** 2025-05-19

**Authors:** Zheng Ye, Qiaoshuang Lu, Dihu Lv, Chun Yang

**Affiliations:** 1 Shanxi Institute for Functional Food, Shanxi Agricultural University, Taiyuan 030001, China; yezheng@sxau.edu.cn; 2 College of Food Science and Engineering, Shanxi Agricultural University, Jinzhong 030801, China; z20223035@stu.sxau.edu.cn (Q.L.); lvdihu@163.com (D.L.)

**Keywords:** hawthorn pectin, response surface methodology, pectin yield optimization, structural characterization, rheological properties, antioxidant activity

## Abstract

This study employed response surface methodology to optimize the conditions for ultrahigh-pressure-assisted enzymatic extraction (UHPEE) of pectin from hawthorn using cellulase. The effects of this method on the characteristics of the extracted pectin were investigated. The optimal extraction parameters were determined to be a solid-to-liquid ratio of 1:70 g/mL, an extraction pressure of approximately 300 MPa, and a holding time of roughly 600 s, yielding a pectin recovery of 4.02%. The optimized UHPEE process resulted in reductions in both the degree of esterification and molecular weight of the pectin, while concurrently increasing the content of total galacturonic acid and total polyphenols. Ion chromatography analysis identified five monosaccharides in the hawthorn pectin, with galacturonic acid being the most predominant. Fourier-transform infrared spectroscopy (FT-IR) and scanning electron microscopy (SEM) analyses revealed the presence of characteristic absorption peaks of pectin and a rough surface topology with a loose, flaky structure, respectively. Rheological measurements demonstrated that the hawthorn pectin exhibited shear-thinning behavior, characteristic of a pseudoplastic fluid. In vitro antioxidant assays showed that hawthorn pectin scavenged 1,1-diphenyl-2-picrylhydrazyl (DPPH) radicals with a rate of 92.72%, comparably to vitamin C at the same concentration (96.30%). These results indicate that the optimized UHPEE method is a more efficient technique for extracting hawthorn pectin and effectively enhances its antioxidant activity, suggesting its potential application in the food industry.

## 1. Introduction

Hawthorn (*Crataegus pinnatifida* Bunge), belonging to the Rosaceae family and the genus Crataegus [1,2], is a plant of significant value in both medicinal and edible fields [3]. Hawthorn is widely cultivated in northern China, renowned for its rich nutritional value and various health benefits, including improved appetite [4], strengthened spleen function, nourishment and moistening, dredging collaterals, and activating blood circulation. The global demand for pectin is substantial. Currently, the primary commercial sources of pectin are citrus peel waste and apple pomace. However, hawthorn contains up to 9% pectin, ranking among the highest in fruits [5], and possesses various biological activities, such as antioxidant [6], antibacterial [7], lipid-lowering [8], and hypoglycemic effects [9]. Hawthorn pectin extracted under optimized conditions contains higher levels of phenolic compounds and has a lower molecular weight [10], which helps to enhance the gelling properties of pectin and its potential health benefits. Therefore, hawthorn is an ideal source for pectin production [11]. However, due to the complex composition of hawthorn pectin [12], its properties and structure are susceptible to the influence of raw materials and extraction processes [13,14], resulting in lower reproducibility of its biological activities [15]. Currently, the extraction methods of hawthorn pectin mainly include hot water extraction, ultrasonic-assisted extraction, microwave-assisted extraction, and deep eutectic solvent extraction, among others. [16]. These methods have their advantages and disadvantages. For example, the water extraction method is simple to operate and has low cost, but the yield of pectin is relatively low [17,18]; ultrasound-assisted extraction can improve the extraction rate [19], but it may lead to a decrease in the activity of pectin polysaccharides [20]; microwave-assisted extraction can shorten the process time and improve the purity of pectin, but the wavelength and power parameters are difficult to control, which affects the quality of pectin [21]; and deep eutectic solvent extraction is a green and environmentally friendly extraction method [22], but for raw materials with high pectin content, such as hawthorn, the extraction process may be affected by increased system viscosity [23], which reduces solvent penetration and thus impacts extraction efficiency.

As a novel nonthermal extraction technology [24], ultrahigh-pressure technology (UHP) has significant advantages in the extraction of natural products. It can disrupt cell structure and improve cell permeability, thereby increasing the yield of extracts [25]. In addition, the reduced extraction time and lower temperature associated with UHP, as compared with hot water extraction (WE), effectively maintain the nutritional value and biological activity of the extracts. Consequently, this diminishes the detrimental effects of thermal damage on the quality of natural products [26,27]. Zhao et al. [28] indicated that using UHP to extract wedelolactone and isoingenylwedelolactone from *Eclipta prostrata* reduced the extraction time from 60 min to 3 min. Chen et al. [29] also confirmed that using UHP to assist in the extraction of polysaccharides from red dragon fruit peels increased the extraction rate by 93.45% compared with heat reflux extraction.

Additionally, the bioenzymatic extraction method is favored by researchers because of its high efficiency, safety, and environmental friendliness [30]. Wei et al. [31] reported that the yield of pectin extracted from papaya peels using the compound enzyme method was 3.26% higher than that reported by Maran et al. [32] using the microwave method. In our previous studies, the yield of hawthorn pectin extracted by cellulase was significantly higher than that extracted by pectinase and xylanase. Nevertheless, the sole use of enzymatic extraction may be associated with issues such as prolonged extraction time and a relatively large amount of enzyme required [33]. In recent years, the combination of UHP and enzymatic extraction has demonstrated potential advantages in the extraction of polysaccharides [29]. This combined technique leverages the strengths of both UHP and bioenzymatic extraction to achieve a more efficient and environmentally friendly extraction of pectin. However, current research on the application of UHP-assisted enzymatic extraction in pectin extraction is still relatively scarce, particularly for pectin derived from specific plant sources, such as hawthorn pectin.

This study is the first to apply UHP-assisted cellulase extraction technology to the extraction of hawthorn pectin, aiming to explore the impact of this technology on the extraction yield and functional properties of hawthorn pectin. It is expected that this will provide an efficient and environmentally friendly new method for preparing high-activity natural pectin, providing a theoretical basis and technical support for its application in the food industry.

## 2. Results

### 2.1. Optimization of the Process for Extracting Hawthorn Pectin

#### 2.1.1. Single Factor Experiment Results

**Figure 1:**
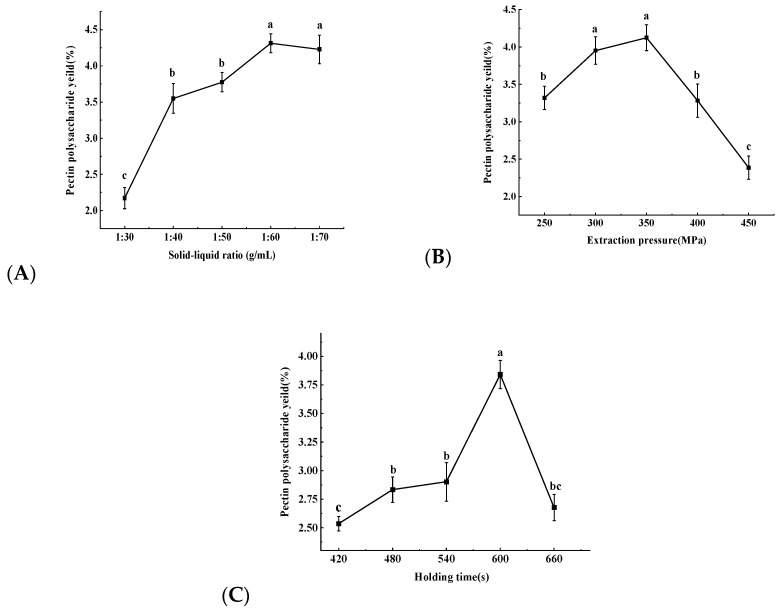
Effects of different liquid–solid ratios (**A**), extraction pressures (**B**), and holding time (**C**) on the extraction efficiency of hawthorn pectin. Different letters indicate significant differences within groups (*p* < 0.05).

It can be seen from Figure 1A that with an increasing solid–liquid ratio, the yield of hawthorn pectin polysaccharides showed an upward trend and gradually leveled off. This indicates that as the volume of the solution increased, it allowed for sufficient contact between the material and the solution, which was beneficial for the release of pectin polysaccharides. When the material-to-liquid ratio was 1:60 g/mL, the yield of pectin polysaccharides in the extraction solution reached the highest value of 4.31%, showing a significant difference compared with other material-to-liquid ratios. However, there was no significant difference compared with the 1:70 g/mL ratio. This may be because the raw material has been sufficiently wetted by the solvent at this point, and almost all the pectin polysaccharides in the hawthorn powder had been dissolved [34]. Therefore, the three levels of 1:50, 1:60, and 1:70 g/mL for the solid–liquid ratio were chosen for the response surface methodology experiment to extract hawthorn pectin polysaccharides.

As shown in Figure 1B, with continuously increasing extraction pressure, the yield of hawthorn pectin polysaccharides showed a trend of first increasing and then decreasing. This may have been due to the increase in pressure in a short period leading to a large pressure difference between the inside and outside of the cells, making the cell walls easier to break down, resulting in a large amount of hawthorn pectin polysaccharides dissolving out [35] and the yield gradually increasing. At an extraction pressure of 350 MPa, the yield of hawthorn pectin polysaccharides reached a maximum value of 4.12%; When the UHP exceeded 350 MPa, the excessive penetration power on the raw material may have led to the extraction of impurities other than pectin polysaccharides, which would have affected the dissolution of pectin polysaccharides, resulting in a rapid decrease in yield [36]. Therefore, extraction pressures of 300, 350, and 400 MPa were selected for further response surface methodology experiments.

As shown in Figure 1C, with the continuous extension of the holding pressure time, the yield of hawthorn pectin polysaccharides showed a trend of first increasing and then decreasing. This was because extending the holding pressure time increased the contact time between the material and the solution, allowing the pectin polysaccharides to gradually dissolve out, thereby continuously increasing the yield of hawthorn pectin polysaccharides. When the holding pressure time reached 600 s, the yield of hawthorn pectin polysaccharides reached a maximum value of 3.84%, which was significantly different from that at other holding pressure times. When the holding pressure time exceeded 600 s, the yield of hawthorn pectin polysaccharides dropped sharply. This may have been because with the prolonged holding pressure time, some of the galacturonic acid in the pectin polysaccharides decomposed more rapidly [37]. It is also possible that because the material particles were small, in the case where the mass transfer process of the solute reached equilibrium and continued for some time, the solute was easily attached to the surface of the material [38], thereby causing a decrease in the yield of hawthorn pectin polysaccharides. Therefore, the holding pressure times of 540, 600, and 660 s were selected for further response surface methodology (RSM) experiments.

#### 2.1.2. Response Surface Analysis

The design and results of the response surface methodology experiments are shown in Table 1. Using the Design–Expert 12 software, a regression analysis and polynomial fitting were performed on the experimental results in Table 1, resulting in the following regression model equation for the yield of hawthorn pectin:

Y = 2.86 + 0.3452A − 0.4650B + 0.2606C − 0.0319AB + 0.1366AC + 0.0856BC + 0.3378A^2^ − 0.0432B^2^ − 0.0508C^2^ (1)

To verify the validity of the regression equation and the degree of influence of each factor on the yield of hawthorn pectin, an analysis of variance (ANOVA) was conducted on the model, and the results are presented in Table 2. In Table 2, in the response surface regression mode, *p* < 0.01 indicates an extremely significant level. The *p*-value for the lack of fit was 0.5875, which was greater than 0.05, indicating that it was not significant. This demonstrates that the established quadratic regression model was valid. The correlation coefficient of the model, R^2^ = 0.9908, and the corrected coefficient of determination, R_adj_^2^ = 0.9789, indicated that 97.89% of the variation in the response values could be explained by this model, indicating a high degree of fit. The coefficient of variation (C.V.) was 2.40%, indicating that the experimental error was small and the reliability was high. Thus, this model demonstrated utility for the experimental design of extracting hawthorn pectin. Further research is warranted to evaluate its applicability to other Crataegus species, as species-specific variations may influence the extraction process.

Based on the F-values of the three factors, the order of their influence on the yield of hawthorn pectin was extraction pressure (B) > liquid-material ratio (A) > holding time (C), and all had a highly significant impact on the yield of hawthorn pectin. BC were significant factors, while AC and A^2^ were highly significant factors, indicating that there was a clear mathematical relationship between these significant and highly significant factors and the yield of hawthorn pectin.

**Figure 2:**
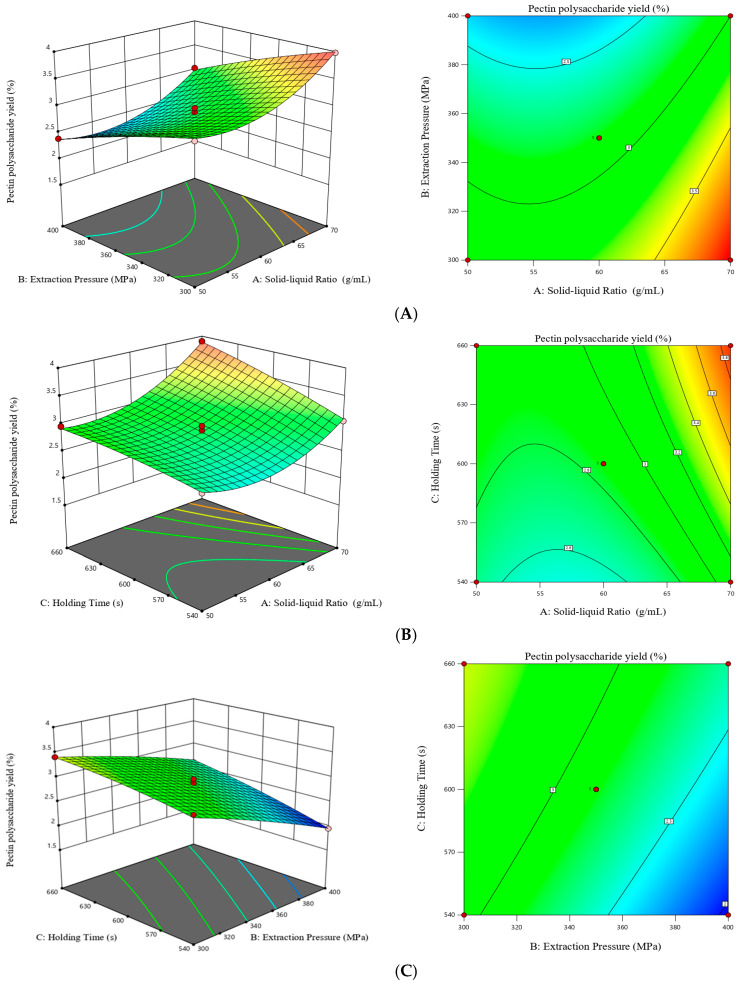
Response surfaces and contour maps of different interaction factors on yield of hawthorn pectin. solid–liquid ratio and extraction pressure (**A**), solid–liquid ratio and holding time (**B**), extraction pressure and holding time (**C**). The different color of balls represent the position of the response value for each factor’s intermediate and extreme levels.

There were pairwise interactions among the three groups of factors: solid–liquid ratio and extraction pressure, solid–liquid ratio and holding time, and extraction pressure and holding time. The response surface and contour plots for the effects of these factors on the yield of hawthorn pectin are shown in Figure 2. As can be seen from the figure, the interaction between the solid–liquid ratio and holding time had the most significant effect on the yield of hawthorn pectin. When the extraction pressure was kept constant, the response value increased with increasing solid–liquid ratio and holding time, and the slope of the response surface was similar, indicating that both the solid–liquid ratio and holding time had significant effects on the yield of hawthorn pectin, which was consistent with the results of variance analysis in Table 2.

Based on the analysis of the response surface methodology experiment, the optimal conditions for the UPAEE were determined to be a material–liquid ratio of 1:69.94 g/mL, an extraction pressure of 302.07 MPa, and a holding time of 604.30 s. The predicted maximum yield was 3.99%. During actual operation, considering the practicality of the experiment, the theoretical process parameters were adjusted to a material-to-liquid ratio of 1:70 g/mL, an extraction pressure of 302 MPa, and a holding time of 604 s. The actual yield of hawthorn pectin obtained was 4.02%. The actual experimental values deviated slightly from the response surface predicted values, but the difference was not significant. Except for possible errors during the experiment, this demonstrates that the regression equation obtained through response surface analysis was reasonable and reliable. It is noteworthy that the pectin extraction yield was significantly lower than the pectin content in hawthorn, likely because of incomplete enzymatic action. Although specific enzymes were used to assist in pectin extraction, their efficiency was affected by factors such as enzyme activity, reaction time, temperature, and pH [39]. Consequently, not all soluble pectin was converted into an extractable form, resulting in some pectin remaining unextracted. Additionally, other losses, such as pectin degradation or mechanical loss, may have occurred during the extraction process.

### 2.2. Characterization Analysis of Properties

#### 2.2.1. Basic Indicators

The basic physicochemical properties of UPAEE, EE, WE, AE, and UPAAE are shown in Table 3. As one of the important physicochemical indicators of pectin, the degree of esterification is related to factors such as the source of pectin, treatment methods, and extraction conditions [40]. The degree of esterification of UPAEE was less than 50%, belonging to low methoxyl pectin, which was significantly different from the esterification degrees of EE, WE, AE, and UPAAE. It should be noted that following the cellulase-assisted extraction (60.43 ± 2.42%), a further extraction using ultrahigh-pressure assistance resulted in a 22.23% reduction in the degree of esterification. This indicates that the ultrahigh-pressure-assisted enzyme treatment reduced the degree of esterification of hawthorn pectin, acting as a de-esterification process. The reason for this phenomenon may be that ultrahigh pressure disrupts the C-O bonds in the carboxyl groups of the pectin chain [41]. This would be consistent with the conclusions drawn by Tian et al. [42] and Zhao et al. [43]. In contrast, the content of total galacturonic acid in UPAEE was higher than that of EE, WE, AE, and UPAAE, which may have been due to the more thorough destruction of the cell wall under high-pressure environments and instantaneous decompression conditions making it easier for galacturonic acid to leach out [44]. Similar results have also been reported in the literature [45], where a study found that after high-pressure pretreatment of passionfruit, the galacturonic acid content of the extracted pectin increased from 65.49% to 80.94%.

The total polyphenol contents of WE, AE, and UPAAE were all below 1%, while the total polyphenol content of UPAEE was 2.16 ± 0.03%, significantly higher than that of the hawthorn pectin extracted by the above three different methods. The reason for this may have been that the ultrahigh-pressure-assisted enzyme treatment disrupted the hydrophobic bonds on the cell membrane, increased the mass transfer rate, and enhanced the retention rate and activity of antioxidant compounds, thereby making phenolic compounds more easily leached from the cells [46]. Zhao et al. [47] also reached the same conclusion, indicating that the use of ultrahigh pressure for extraction can indeed improve antioxidant effects. Compared with hawthorn pectin extracted by EE (622.67 kDa), the molecular weight of that extracted by UPAEE was significantly reduced. The reason may have been that based on cellulase degrading the pectin chain and converting large molecular pectin into small molecules, ultrahigh pressure was applied, and the larger pressure caused the pectin neutral side chains to break, consequently leading to a reduction in molecular weight [48].

**Table 3:** The basic index of UPAEE, EE, WE, AE and UPAAE.

Basic Indicators	UPAEE	EE	WE	AE	UPAAE
Degree of esterification/%	38.20 ± 1.49 ^c^	60.43 ± 2.42 ^a^	56.90 ± 2.44 ^b^	58.42 ± 3.97 ^a^	55.84 ± 2.12 ^b^
Total galacturonic acid content/%	58.09 ± 0.64 ^a^	55.81 ± 1.22 ^b^	55.83 + 0.90 ^b^	52.57 + 0.74 ^c^	53.28 + 0.44 ^c^
Total polyphenols/%	2.16 ± 0.03 ^a^	1.02 ± 0.02 ^b^	0.96 + 0.03 ^c^	0.98 + 0.04 ^c^	0.92 + 0.02 ^d^
Molecular weight/kDa	121.34 ^e^	622.67 ^b^	664.98 ^a^	508.36 ^d^	558.45 ^c^

Note: The pectin esterification degree, total galacturonic acid content, and total polyphenol content of WE, AE, and UPAAE were all from the research team’s previous experimental results [49]. Different letters indicate significant differences within groups (*p* < 0.05).

#### 2.2.2. Monosaccharide Composition

**Figure 3:**
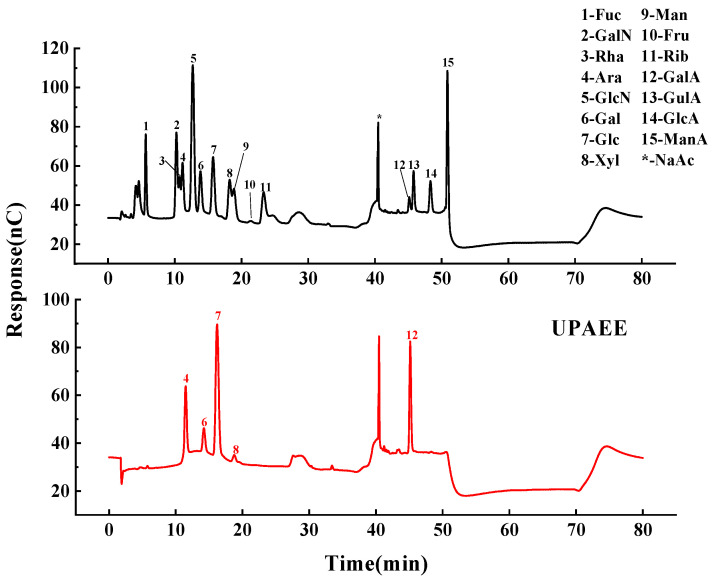
Ion chromatogram of monosaccharide mixed standard and UPAEE.

The ion chromatograms of 15 monosaccharide standards and UPAEE are shown in Figure 3. UPAEE contained five monosaccharides, namely arabinose, galactose, glucose, xylose, and galacturonic acid. Among them, galacturonic acid has the highest content with a molar ratio of 61.3%, indicating that GalA is the main component of UPAEE. Concurrently, a significant increase in the content of GalA was observed in the UPAEE compared with the 25.7% molar ratio found in EE-HP (*p* < 0.5). Furthermore, glucose was identified as the predominant neutral sugar in UPAEE, accounting for 22.2%, followed by arabinose at 10.8%. The presence of higher glucose content, as explained in the literature, may be attributed to the adherence of nonpectic polysaccharides, such as cellulose and hemicellulose, to the pectin side chains [50].

The structure of pectin is mainly composed of three regions: the smooth region of homogalacturonan (HG) and the hairy regions of rhamnogalacturonan I (RG-I) and rhamnogalacturonan II (RG-II) [51]. According to the literature [52], the formulas HG (%) = GalA (%) − Rha (%) and RG-I (%) = 2 Rha (%) + Ara (%) + Gal (%) are used to calculate the corresponding percentages. Based on these formulas, the HG content of UPAEE was calculated to be 61.3%, and the RG-I content was 14.8%. This indicates that the pectin extracted from hawthorn had a core structure composed of a high proportion of HG and a low proportion of RG-I. This result is consistent with findings on pectin extracted from citrus peel using cold water [53]. In addition, the Rha/GalA ratio reflects the proportion of RG-I in the pectin chain, which is used to characterize the number of side chains. If the Rha/GalA ratio is between 0.05 and 1, this indicates that the structure of the pectin is mainly of the RG-I type. When Rha/GalA < 0.05, the pectin structure is mainly composed of HG and RG-II. Since Rha was not detected in UPAEE, it can be concluded that the structure of UPAEE was mainly composed of HG and RG-II. The (Ara + Gal)/Rha ratio reflects the proportion of side chain content in the RG-I domain [54]. The ultrahigh pressure treatment also led to the degradation of pectin side chains, which was consistent with the research findings of Xie et al. [55].

#### 2.2.3. FT-IR

**Figure 4:**
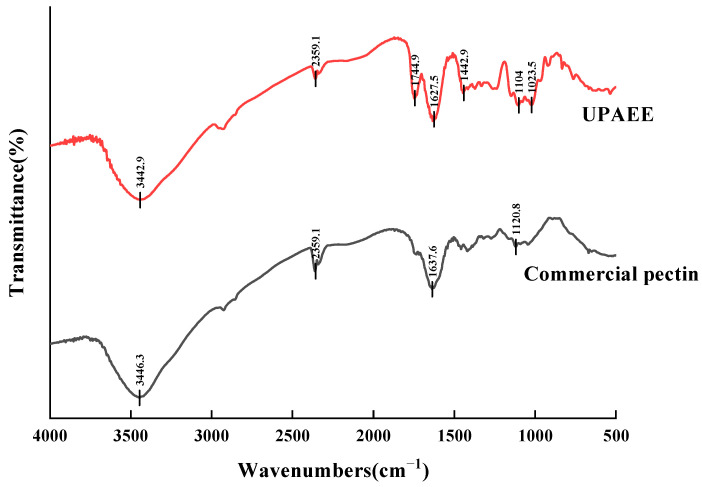
Infrared spectra of UPAEE and commercial pectin.

The infrared spectra of UPAEE and commercial low-ester pectin are shown in Figure 4. From the figure, it can be observed that the absorption peaks of UPAEE were similar to those of commercial pectin, confirming that the extracted substance was low-ester pectin. A broad absorption peak appeared between 3700 and 3000 cm^−1^, which was due to the hydrogen bonding effect between hydrogen and oxygen atoms in the GalA main chain, both intermolecularly and intramolecularly, leading to the O-H stretching vibration. The broadness of the absorption peak may have been caused by the simultaneous presence of -OH groups both intra- and intermolecularly [55]. The absorption peak located at 2360 cm^−1^ may have been due to the stretching vibration of triple bonds or cumulative double bonds [56].

For pectin, the absorption peaks in the range of 1800~1600 cm^−1^ are crucial for identification and quantification [57]. The absorption at 1744 cm^−1^ was due to the stretching vibration of the carbonyl group (C=O) in methylated carboxyl groups [58], while the absorption peak at 1627 cm^−1^ was attributed to the asymmetric stretching vibration of the carbonyl group (C=O) in free carboxyl groups [59]. The presence of these two absorption peaks further confirmed that UPAEE was a pectin substance and indicated that the pectin composition contained aldonic acid [60]. Additionally, the degree of esterification (DE) of pectin can be measured by calculating the ratio of the peak areas [A_1730_/(A_1730_ + A_1630_)]. From the absorbance intensities at these two peaks in Figure 3, it can be concluded that the DE of UPAEE was less than 50%, which was consistent with the results measured by titration shown in Table 4.

The absorption peak at 1442 cm^−1^ was caused by the weak bending vibrations between C-H bonds [61]. The absorption peak at 1104 cm^−1^ may have been due to the overlapping stretching vibrations of ether and ester bonds, indicating the possible presence of pyranose rings in the hawthorn pectin sample [62,63]. The absorption peak at 1023 cm^−1^ indicated the presence of uronic acids in the hawthorn pectin sample [64], which was consistent with the results depicted in Figure 3 showing the presence of galacturonic acid in the hawthorn pectin sample.

#### 2.2.4. SEM

**Figure 5:**
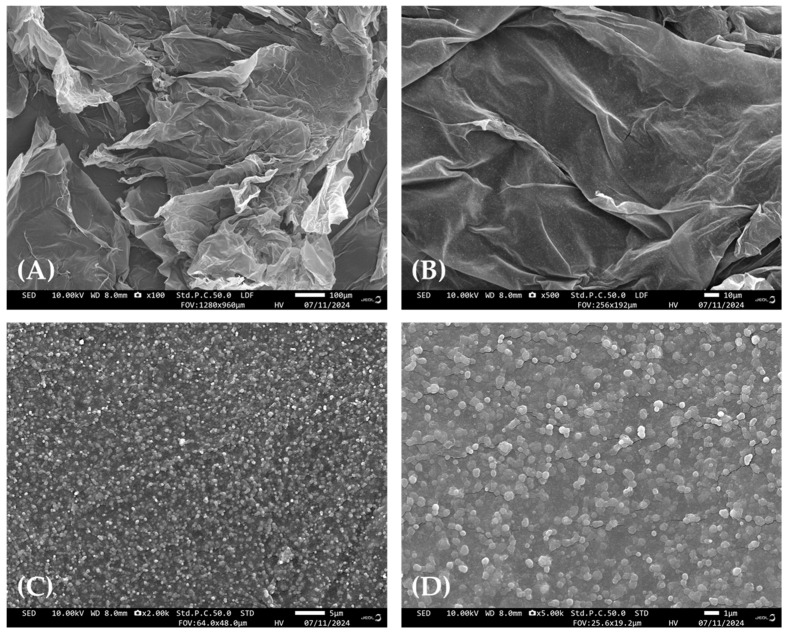
SEM images of UPAEE at magnifications of (**A**–**D**) 100×, 500×, 2000×, and 5000×, respectively.

Scanning electron micrographs of UPAEE at different magnifications are shown in Figure 5. As shown in Figure 5, UPAEE exhibited a loose, flaky structure with a rough surface, and spherical granular substances were attached to it. These substances may have been globular proteins present on their surface [65]. Compared with the SEM images of WE at 100× and 5000× magnification, as previously reported by the experimental team [49], the surface of UPAEE was observed to be prone to curling, with many wrinkles under low magnification. At 5000× magnification, it was observed that the surface had many cracks, indicating the presence of internal voids. This could be attributed to the high extraction pressure and the rapid decompression process.

#### 2.2.5. Rheological Property Analysis

**Figure 6:**
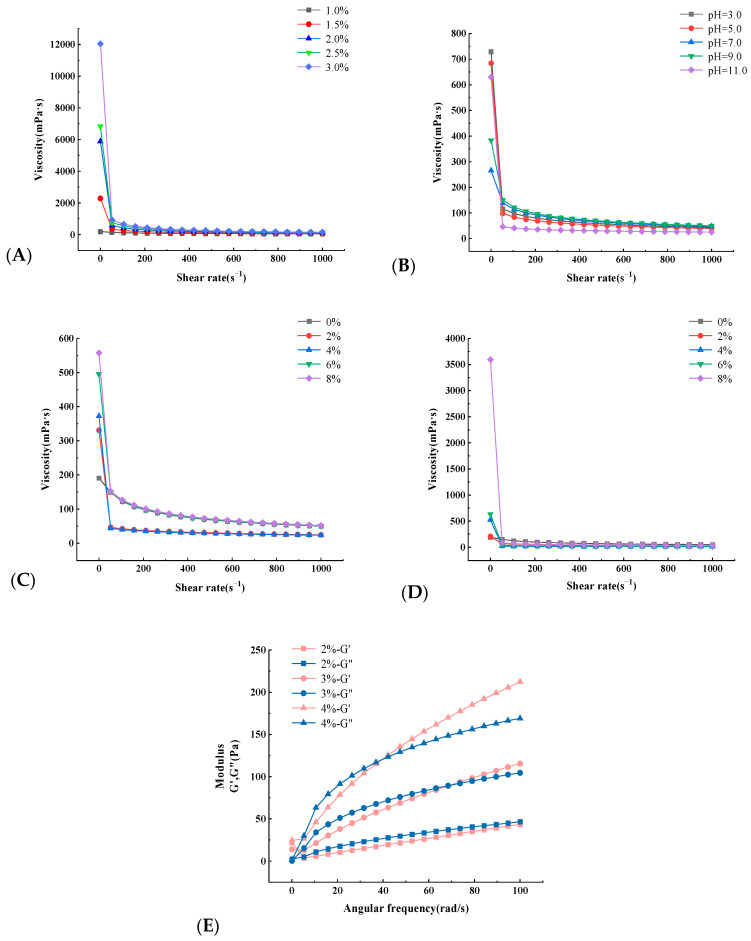
The effects of different mass concentrations (**A**), pH (**B**), Na^+^ concentration (**C**) and Ca^2+^ concentration (**D**) on the shear rheological properties of UPAEE solution; (**E**) Influence of different mass concentrations on G′ and G″ frequency sweep curves of UPAEE solution.

The effects of different mass concentrations on the viscosity of UPAEE solutions were studied within a shear rate range of 0.1–1000 s^−1^. As shown in Figure 6A, the apparent viscosity of UPAEE decreased with increasing shear rate and tended to stabilize, exhibiting the characteristics of shear thinning, which is a typical pseudoplastic fluid in non-Newtonian fluids [66]. The shear thinning may have been the result of the orientation effect. The increase in shear rate causes the pectin molecules to align uniformly in the flow direction, leading to a reduction in the interaction between adjacent pectin chains, thereby gradually decreasing the apparent viscosity and approaching a constant level [67]. Under the same conditions, as the mass concentration increases, the molecules become entangled with each other, forming a more robust network or coiled structure [68], thereby increasing their apparent viscosity. UPAEE exhibited a significantly higher viscosity, indicating that ultrahigh pressure treatment enhanced pectin viscosity. The increased viscosity observed with ultrahigh pressure treatment may be attributed to the higher pressure causing the breakage of pectin molecular side chains, thereby exposing more charges to the solution and consequently increasing the solution’s viscosity [69].

As shown in Figure 6B, under different pH conditions, the viscosity of a 1% UPAEE solution decreased with increasing shear rate, still exhibiting the characteristics of shear thinning as a pseudoplastic fluid. UPAEE exhibited a higher apparent viscosity in solutions at pH 3 and pH 5, indicating that UPAEE had a certain degree of acid resistance. The reason for this may have been that pectin itself is an acidic sugar, and acidic conditions can promote specific intermolecular interactions, such as the formation of hydrogen bonds, leading the chemical structure to be more stable [70]. These interactions would contribute to the formation of a denser network structure, thereby increasing the viscosity of the solution.

During industrial food processing, metal ions are often used as food additives to control the viscosity of food, which can not only improve production efficiency and reduce energy consumption but optimize the performance of the product. Therefore, it is of certain significance to explore the effect of metal ion concentration on the apparent viscosity of hawthorn pectin [71]. The effect of different Na^+^ concentrations on the viscosity of a 1% UPAEE solution is shown in Figure 6C. As shown in the figure, with increasing NaCl mass concentration, the apparent viscosity of UPAEE gradually increased. The reason for this may have been that the charge on the pectin molecules decreased, which enhanced the complexing ability between pectin molecules, forming soluble molecular complexes and thereby increasing the viscosity [72]. It is also possible that by adding Na^+^, the electrostatic repulsion was shielded, enhancing the interactions between pectin chains, thereby promoting the formation of more entanglements and making these interactions more compact [73] and thus increasing the viscosity. Additionally, some studies have shown that adding NaCl to a pectin polysaccharide solution initially increases its viscosity before decreasing it [74], which differed from the results of this study. This also indicates that the effect of NaCl on the apparent viscosity varies depending on the source of the pectin.

Figure 6D shows the changes in viscosity with shear rate for 1% UPAEE solutions at different Ca^2+^ concentrations. From the figure, it can be seen that the UPAEE solution still exhibited the characteristics of a pseudoplastic fluid and that the apparent viscosity of UPAEE still showed a gradual upward trend. When the concentration of Ca^2+^ reached 8%, its viscosity had a very obvious increasing trend. The reason for this may have been that UPAEE contains more -COOH, and with increasing Ca^2+^, cross-linking reactions occurred between the two, thereby enhancing the viscosity of the solution [75]. It should be noted that the amount of Ca^2+^ added needs to be controlled, because excessive Ca^2+^ causes the salting-out effect in the pectin solution, resulting in a decrease in its solution viscosity [76].

The storage modulus (G′) represents the elastic energy that a material can store under the action of an external force, that is, the elastic properties of the material. The loss modulus (G″) represents the energy lost by the material under the action of an external force, that is, the viscous properties of the material. These two parameters can reflect the elastic and viscous properties of the material [77]. Figure 6E shows the relationship between G′ and G″ of UPAEE with different concentration gradients and angular frequencies in the range of 0.1~100 rad/s. From the figure, it can be seen that both G′ and G″ of UPAEE increased with increasing angular frequency, indicating that it had both elasticity and viscosity, which was consistent with the research results of Torralbo et al. [78]. At the same time, both G′ and G″ of UPAEE also increased with increasing pectin concentration. Interestingly, at a certain angular frequency, the G′ curve intersected with the G″ curve for UPAEE of different concentrations. This intersection point is called the crossover frequency, which is the beginning of elastic behavior or the gel state [79]. Therefore, at lower angular frequencies, G′ was lower than G”, and the UPAEE solution exhibited viscous characteristics of a liquid. As the angular frequency continued to increase, after the two curves intersected, G′ became higher than G″, and the elastic characteristics of the solution predominated.

#### 2.2.6. Analysis of Antioxidant Activity

**Figure 7:**
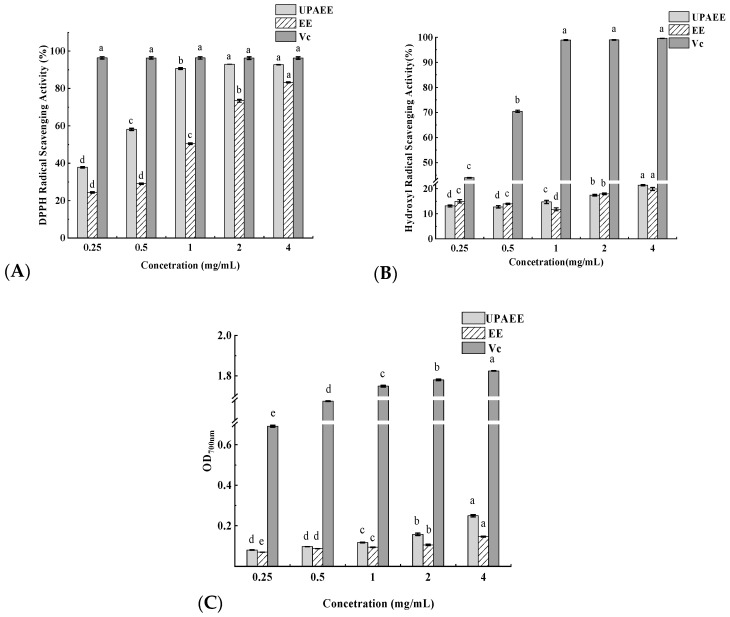
(**A**) Scavenging activity of UPAEE and EE on DPPH free radicals; (**B**) scavenging effect of UPAEE and EE on hydroxyl free radicals; (**C**) total reducing power of UPAEE and EE. Note: different letters indicate significant differences within groups (*p* < 0.05).

The antioxidant effects of UPAEE and EE are shown in Figure 7. UPAEE exhibited significant DPPH radical scavenging activity in a dose-dependent manner. At a concentration of 4 mg/mL, the DPPH scavenging capacities of UPAEE and Vc were 92.72 ± 0.07% and 96.30 ± 0.64%, respectively. UPAEE showed a 9.47% higher DPPH scavenging capacity (92.72 ± 0.29%) than EE (83.25 ± 0.29%). This suggests that ultrahigh pressure extraction enhanced the antioxidant activity of the pectin, a result consistent with the Zheng et al. study [26] on ultrahigh-pressure-assisted extraction of porphyran. Additionally, according to the literature [80], both increased galacturonic acid content and decreased molecular weight can enhance the DPPH scavenging effect of pectin. This is because the reduction in the relative molecular weight of pectin molecules results in a larger surface area for contact with the reducing hydroxyl terminals [81]. Therefore, the ability to scavenge DPPH free radicals is also stronger, which is consistent with the results of this study.

As demonstrated in Figure 7B, the scavenging capacities of UPAEE and EE for hydroxyl radicals exhibited positive correlations with their concentration. Similarly, at a concentration of 4 mg/mL, the scavenging ability of UPAEE for hydroxyl radicals was enhanced in comparison with that of EE (19.90 ± 0.59%). This can be attributed to the higher galacturonic acid content of UPAEE, which has been shown in the literature to accelerate the release of hydrogen atoms on the hydroxyl group and to supply hydrogen for the scavenging of hydroxyl radicals, thereby enhancing their scavenging ability [82]. As demonstrated in the extant literature [83], the synergistic effect of UHP with enzymes results in the weakening of hydrogen bonding strength between and within pectin molecules, the enhancement of hydroxyl capacity, and an increase in the number of hydrogens, thereby improving the scavenging activity against hydroxyl radicals.

As shown in Figure 7C, there was a significant dose–effect relationship between the total reducing power of UPAEE and its concentration. At a concentration of 4 mg/mL, the total reducing power of EE had an absorbance value of 0.147 at 700 nm, whereas that of UPAEE at the same concentration was 0.250, which indicated that the intervention of UHP could enhance its total reducing power, which might be related to the reduction in the esterification degree of UPAEE. Previous studies by others [84] have also shown a negative correlation between the DE value of pectin molecules and their antioxidant activity, and this assay further suggests that lower DE values are likely to be one of the reasons for the increased total reducing power.

## 3. Materials and Methods

### 3.1. Reagents and Instruments

Fresh hawthorn (variety: Dajinxing) was procured in Nanma Village, Xiaodian District (Taiyuan, China) in early October 2022, with a moisture content of 79%. The main reagents included 20,000 U/g cellulase, sourced from Trichoderma reesei, Shandong Longkete Enzyme Preparation Co., Ltd., Jinan, China; commercial low-ester pectin, Xinjiang Fufeng Biological Technology Co., Ltd., Urumqi, China; citric acid, m-hydroxybiphenyl, chloroform, n-butanol, sodium hydroxide, borax, concentrated sulfuric acid, carbazole, phenolphthalein, sodium chloride, and Folin phenol were all purchased from Shanghai Yuanye Technology Co., Shanghai, China; D-(+)-galacturonic acid, gallic acid, and monosaccharide standards were all of chromatographic grade, Shanghai Yuanye Biotechnology Co., Ltd., Shanghai, China; dialysis bags, Hunan Yibo Biotechnology Co., Ltd., Changsha, China.

The main equipment was a SCIENTZ-10YD/A Freeze Dryer, Ningbo Xinzhi Freeze Dry Equipment Co., Ltd., Ningbo, China; an FW100 High-Speed Grinder, Tianjin Taisite Instrument Co., Ltd., Tianjing, China; a PHS-3C pH Meter, Shanghai Yidian Scientific Instrument Co., Ltd., Shanghai, China; a DZKW-4 Electronic Constant Temperature Water Bath, Beijing Zhongxing Weiye Instrument Co., Ltd., Beijing, China; an SHPP-8.8 L-600 MPa Ultrahigh Pressure Equipment, Shanxi San Shui He Technology Co., Ltd., Taiyuan, China; an LXJ-IIB Low-Speed Centrifuge, Shanghai Anting Scientific Instrument Factory, Shanghai, China; an SHA-CA Water Bath Constant Temperature Shaker, Changzhou Longyue Instrument Manufacturing Co., Ltd., Changzhou, China; an LC-10A High Performance Liquid Chromatograph, Shimadzu Corporation, Tokyo, Japan; a Nicolex IS5 Fourier Transform Infrared Spectrometer (Spectral range 7800–350 cm^−1^, resolution better than 0.8 cm^−1^) and an ICS5000 Ion Chromatograph, Thermo Fisher Scientific Inc., Shanghai, China; JSM-7500F Scanning Electron Microscope (cold field emission scanning electron microscope), JEOL, Tokyo, Japan; and an MCR302 Rheometer, Anton Paar, Graz Austria.

### 3.2. Raw Material Pretreatment

Fresh hawthorns, uniform in size and free from pests and diseases, were thoroughly cleaned and immersed in boiling water for 1 min (to inactivate enzymes). They were then rapidly removed, cooled, cored, and chopped. The resulting pieces were evenly spread on a metal tray and frozen overnight at −40 °C. Subsequently, the frozen material was subjected to vacuum freeze-drying for 36 h. The dried material was then ground into a fine powder using a high-speed grinder and passed through a 60-mesh sieve. Finally, the powder was placed in a sealed bag and stored in a refrigerator for subsequent extraction.

### 3.3. Extraction and Purification of Hawthorn Pectin

UPAEE: A certain amount of well-mixed hawthorn powder and 0.75% cellulase were weighed and placed into a durable, resilient, and airtight plastic bottle with appropriate capacity. Distilled water with a pH of 4 (adjusted with 2 mol/L citric acid solution) was added according to the specified solid-to-liquid ratio, and the mixture was thoroughly shaken to ensure homogeneity. The bottle was then placed in a 35 °C water bath and extracted for 40 min. After extraction, the mixture was immediately subjected to ultrahigh-pressure processing for a second extraction, with the extraction pressure and holding time set according to the experimental design, temperature 20 °C. The pressure was released instantaneously, and the mixture was then placed in boiling water for 15 min to inactivate enzymes. The extraction solution was centrifuged at 5000 rpm for 20 min, and the supernatant was collected for vacuum concentration. The concentrated solution was mixed with one-fourth volume of Sevage reagent (chloroform–n-butanol = 4:1) and shaken at 300 rpm for 20 min using an oscillator. The mixture was then allowed to stand at room temperature for 20 min and subsequently centrifuged at 5000 rpm for 10 min. The deproteinization process using Sevage reagent was repeated twice. The solution was then dialyzed for 48 h. Four volumes of anhydrous ethanol were added, and the solution was placed at 4 °C overnight. After centrifugation, the supernatant was discarded, and the precipitate was redissolved. The solution was then subjected to vacuum freeze-drying for 24 h to obtain hawthorn pectin, which was weighed and designated as UPAEE.

EE: A certain amount of mixed hawthorn powder and 0.75% cellulase were weighed and mixed according to the solid–liquid ratio, and distilled water with a pH of 4 was added. The mixture was adjusted to the corresponding pH value using a 2 mol/L citric acid solution, shaken thoroughly, and then placed in a constant temperature water bath for extraction. After extraction, the mixture was subjected to enzyme deactivation in a boiling water bath for 15 min. The subsequent steps were consistent with those of the UHP-assisted enzymatic extraction method, and the obtained pectin was named EE.

### 3.4. Single-Factor and Response Surface Methodology Experiments

#### 3.4.1. Single-Factor Experimental Design

The UHP-assisted enzymatic extraction process of pectin from hawthorn was investigated. Single-factor experiments were carried out to establish the feasible ranges for material-to-liquid ratio, extraction pressure, and holding time. These experiments provided fundamental data for subsequent RSM analysis and examined the influence of each factor on pectin yield. The influence of the material-to-liquid ratio (1:30, 1:40, 1:50, 1:60, 1:70 g/mL) on the polysaccharide yield of hawthorn pectin was investigated with the extraction pressure fixed at 350 MPa and the holding time fixed at 540 s. The effect of extraction pressure (250, 300, 350, 400, 450 MPa) on the polysaccharide yield of hawthorn pectin was evaluated with the material-to-liquid ratio fixed at 1:50 g/mL and the holding time fixed at 540 s. The impact of holding time (420, 480, 540, 600, 660 s) on the polysaccharide yield of hawthorn pectin was assessed with the material-to-liquid ratio fixed at 1:50 g/mL and the extraction pressure fixed at 350 MPa.

#### 3.4.2. RSM

Based on the results of the single-factor experiments and utilizing the Design–Expert 12 software, a Box–Behnken design from RSM was employed to optimize the extraction conditions. The material-to-liquid ratio, extraction pressure, and holding time were selected as the influencing factors, with each factor set at three levels. A total of 17 experiments were conducted, including five replicate experiments at the center points to evaluate experimental error and fit the model. The yield of pectin polysaccharides was used as the response value, and the coded levels for each factor are shown in Table 4.

The material-to-liquid ratio, extraction pressure, and holding time were selected as influencing factors. A three-factor, three-level analytical approach was employed to optimize the extraction process of polysaccharides from hawthorn fruit pectin. The yield of pectin polysaccharides was used as the response value. The specific factors and levels are detailed in Table 4.

#### 3.4.3. Calculation of Hawthorn Pectin Yield

A standard solution of D-Gal A with a concentration of 100 μg/mL was prepared. Aliquots of 0, 0.05, 0.10, 0.15, 0.20, and 0.25 mL of the standard solution were transferred into six 10 mL stoppered test tubes, labeled as 0, 1, 2, 3, 4, and 5, respectively. Each tube was supplemented with distilled water to a total volume of 0.25 mL. Then, 1.5 mL of 0.0125 mol/L sodium tetraborate–sulfuric acid solution was added to each tube, and the contents were mixed thoroughly using a vortex mixer after all additions were completed. The tubes were incubated in an 80 °C water bath for 6 min and then cooled to room temperature in ice water. To each tube, 25 μL of m-hydroxybiphenyl solution (0.15%) was added and mixed well, followed by a 20 min incubation at room temperature. The absorbance was measured at 520 nm using the tube labeled 0 as the blank for zero adjustment. A standard curve was plotted with the concentration of D-Gal A standard solution on the x-axis and the absorbance on the y-axis.

A dried sample powder weighing 0.001 g was dissolved in distilled water and made up to a final volume of 10 mL. A 1.0 mL aliquot of the 0.1 mg/mL sample solution was transferred into a 20 mL stoppered test tube. The subsequent steps were carried out as per the standard curve procedure to determine the absorbance. The content of hawthorn pectin was calculated using the standard curve equation. The yield of hawthorn pectin was calculated using the following formula:

X = (m_1_ × c)/m (2)

where X: yield of hawthorn pectin (%); m: the mass of the hawthorn powder used (g); m_1_: the mass of the extracted hawthorn pectin (g); c: the content of D-Gal A calculated based on the concentration derived from the standard curve (%).

### 3.5. Basic Indices

#### 3.5.1. Degree of Esterification (DE) of Pectin

DE reflects the extent to which the methyl groups in pectin molecules are esterified into methoxyl groups, and it is an important indicator for characterizing the properties of pectin.

Of the pectin sample, 0.25 g was weighed and dissolved thoroughly in 50 mL of distilled water at 40 °C. To 10 mL of the 5 mg/mL sample solution was added three drops of 10 g/L phenolphthalein solution (1% mass concentration) as an indicator. The mixture was titrated with 0.001 mol/L NaOH solution until the solution turned pink and remained unchanged for 30 s, recording the volume of NaOH solution consumed as V_1_. Then, 2 mL of 0.005 mol/L NaOH solution were added to the mixture, and the mixture was shaken well and allowed to stand at room temperature for 15 min. Titration continued with 0.005 mol/L HCl solution until the pink color just disappeared and remained unchanged for 30 s. Finally, three drops of 10 g/L phenolphthalein were added, and the mixture was titrated with 0.001 mol/L NaOH solution until the solution turned pink and remains unchanged for 30 s, recording the volume of NaOH solution consumed as V_2_. Degree of esterification was calculated according to the following formula:

DE(%) = V_2_/(V_1_ + V_2_) × 100 (3)

where V_1_: the initial volume of NaOH consumed (mL); V_2_: represents the final volume of NaOH consumed (mL).

#### 3.5.2. Determination of Total Galacturonic Acid Content

Galacturonic acid is the most predominant component in pectin, and its content represents the purity of pectin.

An amount of 0.5 mL of D-Gal A standard solution (with concentrations of 20, 40, 60, 80, and 100 mg/L) was added into a stoppered test tube. Under an ice-water bath condition, 3 mL of 5 mg/mL borax–sulfuric acid solution was added, and the result was mixed thoroughly using a vortex mixer. The mixture was incubated in a boiling water bath for 5 min, then removed and cooled immediately. An amount of 0.1 mL of 0.1% carbazole solution was evenly mixed with the mixture; the mixture was boiled in water for 5 min, and after cooling to room temperature, the absorbance at 530 nm was measured. A standard curve was plotted with the concentration of D-Gal A standard solution as the abscissa and the absorbance value as the ordinate. The standard solution was replaced with 0.5 mL of pectin sample solution, the absorbance value was measured, and the total galacutornic acid content was calculated accordingly.

#### 3.5.3. Total Polyphenols

The total polyphenol content in UPAEE was determined using the Folin–Ciocalteu method as described in the literature [9].

To 20 μL of gallic acid standard solution (with concentrations of 40, 50, 80, 120, and 140 mg/L) was added 100 μL of Folin–Ciocalteu phenol reagent (10%). The two were mixed well and reacted for 5 min. Then 80 μL of Na_2_CO_3_ solution and 50 μL of water were added, and the mixture was allowed to stand at room temperature for 60 min. Of the mixture, 200 μL was placed in a microplate. A microplate reader was used to measure the absorbance at 765 nm. A standard curve was plotted with the concentration of the gallic acid standard solution as the abscissa and the absorbance value as the ordinate. The standard solution was replaced with 20 μL of the sample solution to measure the absorbance value, and the result was calculated.

#### 3.5.4. Molecular Weight Distribution

The molecular weight of pectin is an important factor affecting its properties. The molecular weight of the hawthorn pectin sample was determined using high-performance gel permeation chromatography (HPGPC), equipped with a series of gel columns and a differential refractive index detector.

Preparation of standard solution: A total of 5 mg of the standard (dextran) was accurately weighed and dissolved in 1 mL of mobile phase solution to prepare a 5 mg/mL solution. The sample was transferred to a 1.8 mL sample vial.

Preparation of sample solution: A total of 5 mg of the sample was accurately weighed and dissolved in 1 mL of mobile phase solution to prepare a 5 mg/mL solution. After ultrasonication for 10 min, the solution was centrifuged at 12,000 rpm for 10 min. The supernatant was withdrawn, filtered through a 0.22 μm aqueous microporous membrane, and then transferred to a 1.8 mL sample vial.

A 0.05 M NaCl solution was used as the mobile phase. The lyophilized hawthorn pectin sample was dissolved in the mobile phase to a concentration of 5 mg/mL, subjected to ultrasonication for 10 min, and then centrifuged. The supernatant was filtered through a 0.22 μm aqueous microporous membrane and then placed in a sample vial for analysis. The injection conditions were as follows: injection volume of 25 μL, column temperature of 40 °C, flow rate of 0.8 mL/min, and elution time of 60 min. Mobile phase: 0.05 M NaCl solution; column: BRT105-103-101 series gel column (8 × 300 mm); flow rate: 0.8 mL/min; column temperature: 40 °C; injection volume: 25 μL; detector: RID-20A refractive index detector; analysis time: 60 min.

### 3.6. Structural Characterization

#### 3.6.1. Monosaccharide Composition

The monosaccharide composition of UPAEE was determined using an ion chromatograph.

Fifteen monosaccharides, including fucose (Fuc), galactosamine hydrochloride (GalNl), rhamnose (Rha), arabinose (Ara), glucosamine hydrochloride (GlcN HCl), galactose (Gal), glucose (Glc), xylose (Xyl), mannose (Man), fructose (Fru), ribose (Rib), galacturonic acid (GalA), guluronic acid (GulA), glucuronic acid (GlcA), and manuronic acid (ManA), were employed as standards. Each standard was hydrolyzed with 2 mL of trifluoroacetic acid (TFA, 3M) at 120 °C for 3 h. The hydrolysates were dried under a nitrogen stream, reconstituted in deionized water by vortex mixing, and prepared as stock solutions with concentrations of 2.5 mg/L, 1.5 mg/L, 2.5 mg/L, 1.875 mg/L, 2.5 mg/L, 2.5 mg/L, 2.5 mg/L, 2.5 mg/L, 2.5 mg/L, 7.5 mg/L, 5 mg/L, 2.5 mg/L, 5 mg/L, 2.5 mg/L, and 5 mg/L, respectively. Accurately prepared mixtures of these monosaccharide standards served as mixed standards for absolute quantification.

Hawthorn pectin lyophilized samples were hydrolyzed into monosaccharides using 2 mL of trifluoroacetic acid (TFA, 3M) at 120 °C for 3 h within an ampoule bottle. The acid hydrolysates were precisely transferred to a tube and dried under a nitrogen stream. Subsequently, 5 mL of water was added, and the solution was vortex mixed. A 50 µL aliquot of this solution was then diluted with 950 µL of deionized water and centrifuged. The supernatant was analyzed by ion chromatography. The chromatographic conditions were as follows: the column used was a Dionex Carbopac TMPA20 (3150 mm); the mobile phase consisted of A: H_2_O, B: 250 mmol/L sodium hydroxide, and C: 500 mmol/L sodium hydroxide and 50 mmol/L sodium acetate; the flow rate was set at 0.3 mL/min; the injection volume was 25 µL; the column temperature was maintained at 30 °C; the elution gradient program was: 0 min A/B/C (98:2:0, *v*/*v*), 23 min A/B/C (98:2:0, *v*/*v*), 23.1 min A/B/C (80:20:0, *v*/*v*), 33 min A/B/C (80:20:0, *v*/*v*), 33.1 min A/B/C (80:0:20, *v*/*v*), 46 min A/B/C (80:0:20, *v*/*v*), 46.1 min A/B/C (20:0:80, *v*/*v*), 66 min A/B/C (20:0:80, *v*/*v*), 66.1 min A/B/C (98:2:0, *v*/*v*), 80 min A/B/C (98:2:0, *v*/*v*); and the detector used was electrochemical.

#### 3.6.2. FT-IR

FT-IR was employed to scan the UPAEE sample using an FT-IR spectrometer(Thermo Fisher Scientific Inc., Shanghai, China). The freeze-dried hawthorn pectin sample was mixed with potassium bromide in a 1:100 ratio, blended uniformly, and ground. This mixture was then evenly spread on a glass slide and pressed into a transparent pellet without any cracks. Subsequently, the pellet was placed in the sample holder for scanning. The scanning range was set from 4000 to 400 cm^−1^, with a resolution of 4 cm^−1^ and 64 scans.

#### 3.6.3. SEM

SEM was employed to analyze the surface morphology of UPAEE. To maintain consistency in imaging conditions and compare the structure of pectin at different scales, SEM was used at all magnifications in this study. The sample was fixed on a sample stage and subjected to gold sputtering under vacuum conditions. The surface morphology of the pectin was then captured at an accelerating voltage of 10 kV and magnifications of 100×, 500×, 2000×, and 5000×.

### 3.7. Rheological Properties

#### 3.7.1. Viscosity Measurement

The static rheology of UPAEE was explored using the viscosity curve mode of a rheometer, considering different mass concentrations, pH values, and types and concentrations of metal ions. A 1 mL aliquot of the pectin solution was placed on the sensor platform, the measurement temperature was set to 25 °C, and the cone plate CP50-1 was selected. The viscosity changes of UPAEE were observed over a shear rate range of 0.1~1000 s^−1^.

#### 3.7.2. Dynamic Viscoelasticity Measurement

Dry sample powder was formulated into pectin solutions with different concentration gradients (2%, 3%, and 4%), and dynamic rheological studies were conducted using a frequency sweep mode. A 1 mL aliquot of the pectin solution was placed on the sensor platform, the measurement temperature was set to 25 °C, and the cone plate model CP50-1 was selected. The changes in storage modulus (G′) and loss modulus (G″) of UPAEE were observed under a shear strain of 2% and angular frequencies ranging from 0.1 to 100 rad/s. The dynamic viscoelasticity of UPAEE under different conditions was analyzed.

### 3.8. Determination of In Vitro Antioxidant Activity

Using Vc as the control group, the dry sample powder was formulated into sample solutions with different concentration gradients (0.25, 0.50, 1.00, 2.00, and 4.00 mg/mL) for in vitro antioxidant activity experiments.

#### 3.8.1. DPPH Radical Scavenging Capacity

An amount of 4.9 mg of DPPH was weighed into a 50 mL brown volumetric flask, dissolved in anhydrous ethanol, and diluted to the required volume. In the reaction system, 0.14 mL of DPPH-anhydrous ethanol solution was added first, followed by 0.14 mL of pectin solution with different concentration gradients. After thorough mixing, the reaction mixture was placed in the dark at room temperature for 30 min. This process was repeated three times, and then the absorbance value was measured at 517 nm. The DPPH scavenging rate of pectin solutions at each concentration was calculated according to the following formula:

Clearance rate (%) = 1 − (A_c_ − A_d_)/A_0_ (4)

where A_0_: the absorbance value of DPPH· with anhydrous ethanol; A_c_: the absorbance value of the sample solution, DPPH· with anhydrous ethanol; A_d_: the absorbance value of the sample solution with anhydrous ethanol.

#### 3.8.2. Hydroxyl Radical Scavenging Capacity

In sequence, 0.5 mL of 9 mM FeSO_4_ solution, 0.5 mL of 9 mM ethanol–salicylic acid solution, 0.5 mL of pectin samples at different concentration gradients, 3.5 mL of distilled water, and 0.5 mL of 8.8 mM H_2_O_2_ were absorbed into the reaction system. It is important to note that the reaction mixture was added in order. After thorough mixing, the mixture was incubated at 37 °C for 15 min. Then, the mixture was slowly removed from the water bath. Importantly, it was not shaken when being removed, as this could easily mix the precipitate. The absorbance was measured at 510 nm. The hydroxyl radical scavenging rate of each concentration of pectin solution was calculated using the following formula:

Scavenging rate (%) = 1 − (A_c_ − A_d_)/A_0_ (5)

where A_0_: the absorbance of the control reaction (without pectin sample); A_c_: the absorbance of the reaction containing the pectin sample at different concentration gradients; A_d_: the absorbance of the reaction without FeSO_4_ solution.

#### 3.8.3. Total Reducing Power

Add to 0.1 mL of pectin samples with different concentration gradients 0.5 mL of phosphate buffer (pH 6.6) and 0.5 mL of 1% K_3_[Fe(CN)_6_] solution. The mixture was let stand for 20 min at 50 °C; then, 0.5 mL of 10% trichloroacetic acid solution was added, mixed well, and let stand at room temperature for 10 min. To 0.12 mL of the mixture was added 0.12 mL of distilled water and 25 μL of 0.1% FeCl_3_ solution. After 10 min of reaction, the absorbance value was measured at 700 nm. The higher the absorbance value, the stronger the reducing power.

### 3.9. Data Processing

All experiments were repeated three times. The experimental results were analyzed for significance using SPSS 27.0 software. Graphs were plotted using the Origin 2022 software. Response surface methodology was used for the optimization design and analysis of results using Design–Expert 12.

## 4. Conclusions

In this study, UPAEE was employed to extract UPAEE from hawthorn. The extraction process parameters were optimized through single-factor experiments and response surface methodology: a liquid-to-material ratio of 1:70 g/mL, an extraction pressure of 302 MPa, and a holding time of 604 s. Under the actual optimal process conditions, the yield of UPAEE was 4.02%. With the synergistic action of UHP and cellulase, the DE and molecular weight of UPAEE decreased, while the contents of total galacturonic acid and total polyphenols increased to some extent. The monosaccharide composition was dominated by galacturonic acid, and the structure of UPAEE was mainly composed of HG and RG-II. The infrared scanning range contained characteristic absorption peaks of pectin. SEM analysis showed that UPAEE exhibited a loose, flaky structure with a rough surface that was prone to curling and had attached spherical granular substances. Rheological results indicated that UPAEE exhibited pseudoplastic fluid shear-thinning behavior, where its apparent viscosity gradually decreased and stabilized with increasing shear rate. The storage modulus (G′) and loss modulus (G″) of UPAEE were positively correlated with both angular frequency and its solution concentration; UPAEE exhibited dose-dependent antioxidant activity and demonstrates strong antioxidant capacity. In summary, all the tests and analyses demonstrated that UPAEE possesses the advantages of low molecular weight, low esterification degree, high total polyphenol content, and strong antioxidant activity. UPAEE has potential advantages in the fields of food additives and functional foods. This result enriches the production methods of pectin and provides theoretical support for the development of functional foods, as well as enhancing the comprehensive and high-value application of hawthorn fruit resources.

## Figures and Tables

**Table 1:** Box–Behnken design matrix and response values for the yield of hawthorn pectin.

Number	A Solid–Liquid Ratio/(g/mL)	B Extraction Pressure/(MPa)	C Holding Time/(s)	Pectin Polysaccharide Yield/(%)
1	−1	0	−1	2.67 ± 0.10
2	−1	1	0	2.39 ± 0.11
3	−1	−1	0	3.20 ± 0.18
4	−1	0	1	2.96 ± 0.06
5	0	−1	1	3.41 ± 0.02
6	0	1	−1	1.95 ± 0.17
7	0	0	0	2.97 ± 0.08
8	0	0	0	2.76 ± 0.11
9	0	0	0	2.83 ± 0.12
10	0	0	0	2.84 ± 0.10
11	0	0	0	2.89 ± 0.15
12	0	−1	−1	3.11 ± 0.26
13	0	1	1	2.59 ± 0.13
14	1	0	1	3.90 ± 0.11
15	1	−1	0	3.98 ± 0.07
16	1	1	0	3.04 ± 0.22
17	1	0	−1	3.06 ± 0.19

**Table 2:** Analysis of variance of regression model of hawthorn pectin extraction.

Source of Variance	Sum of Squares	Degrees of Freedom	Mean Square	F-Value	*p*-Value	Significance
Model	3.82	9	0.4247	83.48	<0.0001	**
A (Solid–Liquid Ratio)	0.9534	1	0.9534	187.39	<0.0001	**
B (Extraction Pressure)	1.73	1	1.73	339.95	<0.0001	**
C (Holding time)	0.5434	1	0.5434	106.8	<0.0001	**
AB	0.0041	1	0.0041	0.7998	0.4009	
AC	0.0746	1	0.0746	14.67	0.0065	**
BC	0.0293	1	0.0293	5.76	0.0474	*
A^2^	0.4805	1	0.4805	94.43	<0.0001	**
B^2^	0.0078	1	0.0078	1.54	0.2544	
C^2^	0.0109	1	0.0109	2.13	0.1874	
Residual	0.0356	7	0.0051			
Lack of Fit	0.0126	3	0.0042	0.7257	0.5875	
Pure Error	0.0231	4	0.0058			
sum	3.86	16				

Note: ** indicates a highly significant difference (*p* < 0.01); * indicates a significant difference (*p* < 0.05).

**Table 4:** Factors and levels of RSM for extraction of pectin polysaccharides from hawthorn.

Level	A Solid–Liquid Ratio (g/mL)	B Extraction Pressure (MPa)	C Holding Time (s)
−1	1:50	300	540
0	1:60	350	600
1	1:70	400	660

## Data Availability

The original contributions presented in the study are included in the article, further inquiries can be directed to the corresponding author.
